# Activated forms of astrocytes with higher GLT-1 expression are associated with cognitive normal subjects with Alzheimer pathology in human brain

**DOI:** 10.1038/s41598-018-19442-7

**Published:** 2018-01-26

**Authors:** Eiji Kobayashi, Masako Nakano, Kenta Kubota, Nobuaki Himuro, Shougo Mizoguchi, Takako Chikenji, Miho Otani, Yuka Mizue, Kanna Nagaishi, Mineko Fujimiya

**Affiliations:** 1 Department of Anatomy, Sapporo Medical University, School of Medicine, Sapporo, Hokkaido 060-8556 Japan; 2 Department of Physical Therapy, Faculty of Human Science, Hokkaido Bunkyo University, Eniwa, Hokkaido 061-1449 Japan; 3 Department of Physical Therapy, Hokkaido Chitose Rehabilitation University, Chitose, Hokkaido 066-0055 Japan; 4 Department of Public Health, Sapporo Medical University, School of Medicine, Sapporo, Hokkaido 060-8556 Japan

## Abstract

Although the cognitive impairment in Alzheimer’s disease (AD) is believed to be caused by amyloid-β (Aβ) plaques and neurofibrillary tangles (NFTs), several postmortem studies have reported cognitive normal subjects with AD brain pathology. As the mechanism underlying these discrepancies has not been clarified, we focused the neuroprotective role of astrocytes. After examining 47 donated brains, we classified brains into 3 groups, no AD pathology with no dementia (N-N), AD pathology with no dementia (AD-N), and AD pathology with dementia (AD-D), which represented 41%, 21%, and 38% of brains, respectively. No differences were found in the accumulation of Aβ plaques or NFTs in the entorhinal cortex (EC) between AD-N and AD-D. Number of neurons and synaptic density were increased in AD-N compared to those in AD-D. The astrocytes in AD-N possessed longer or thicker processes, while those in AD-D possessed shorter or thinner processes in layer I/II of the EC. Astrocytes in all layers of the EC in AD-N showed enhanced GLT-1 expression in comparison to those in AD-D. Therefore these activated forms of astrocytes with increased GLT-1 expression may exert beneficial roles in preserving cognitive function, even in the presence of Aβ and NFTs.

## Introduction

Alzheimer’s disease (AD) is primarily responsible for dementia, and its dramatic increase in the last decade is a worldwide problem^1^. The neurodegeneration caused by AD is believed to be triggered by an accumulation of amyloid-β (Aβ) plaques and neurofibrillary tangles (NFTs)^2,3^. Aβ plaques induce synaptic dysfunction and neuronal loss due to their toxicity, and also enhance the phosphorylation of tau^4,5^. NFTs are formed by aggregated phosphorylated tau and are involved in neuronal impairment and cell death^6^. Glutamate transmission at synapses is disturbed by the deposition of Aβ and NFTs, and the subsequent elevation in glutamate at axon terminals is associated with cognitive impairment^7^.

Although Aβ plaques and NFTs are hallmarks of AD, several postmortem studies have shown the presence of cognitive normal subjects with AD-specific pathological changes in the brain^8,9^. For example, the Medical Research Council Cognitive Function and Aging Study (MRC CFAS) revealed that 34.7% of subjects with massive neuritic plaques in the neocortex showed no dementia^10^. Further, the Nun study found that 32% of subjects with moderate NFT deposition in the neocortex showed no dementia^11^. The reason why AD-specific pathological changes do not cause cognitive impairment is based on differences in the length of education, occupation and mental status in childhood^12–14^; however, the detailed brain mechanisms involved in these discrepancies remain unclear.

Recently it has been reported that cognitive impaired subjects with an AD-specific pathology show a decreased number of neurons and synapses in the hippocampus and cerebral cortex, while cognitive normal subjects with an AD-specific pathology show a constant number of neurons and synaptic integrity in these brain regions^15,16^. In addition, an increased number of astrocytes or microglia was found in cognitive impaired subjects with an AD-specific pathology^15,16^, while no such increase was apparent in cognitive normal subjects with an AD-specific pathology. Astrocytes play a number of roles in supporting neurons and maintaining synaptic transmission^17^; however, an accumulation of Aβ plaques stimulate astrocyte conversion to a reactive type that release pro-inflammatory cytokines^18^. In contrast to such harmful reactive astrocytes, beneficial reactive astrocytes that play neuroprotective roles or aid in Aβ clearance in the aged brain were also found^19,20^.

We hypothesized that astrocytes in the brain from cognitive normal subjects with an AD-specific pathology might be of the beneficial type that play a neuroprotective role against Aβ and NFT deposition. To clarify this hypothesis, we examined the characteristics of astrocytes in postmortem brains from both cognitive normal and impaired subjects with an AD-specific pathology. The brains were obtained from bodies donated to Sapporo Medical University, and included a variety of cognitive states. We were particularly interested in the amyloid and tau hypothesis as it does not sufficiently explain AD based on the fact that drugs targeting Aβ are not effective in treating cognitive impairment^21^. The results of the present study led us to first propose the brain mechanism involved in maintaining cognitive function despite the progression of AD-specific pathological changes.

## Results

### Classification of AD neuropathological changes and cognitive function

**Figure 1:**
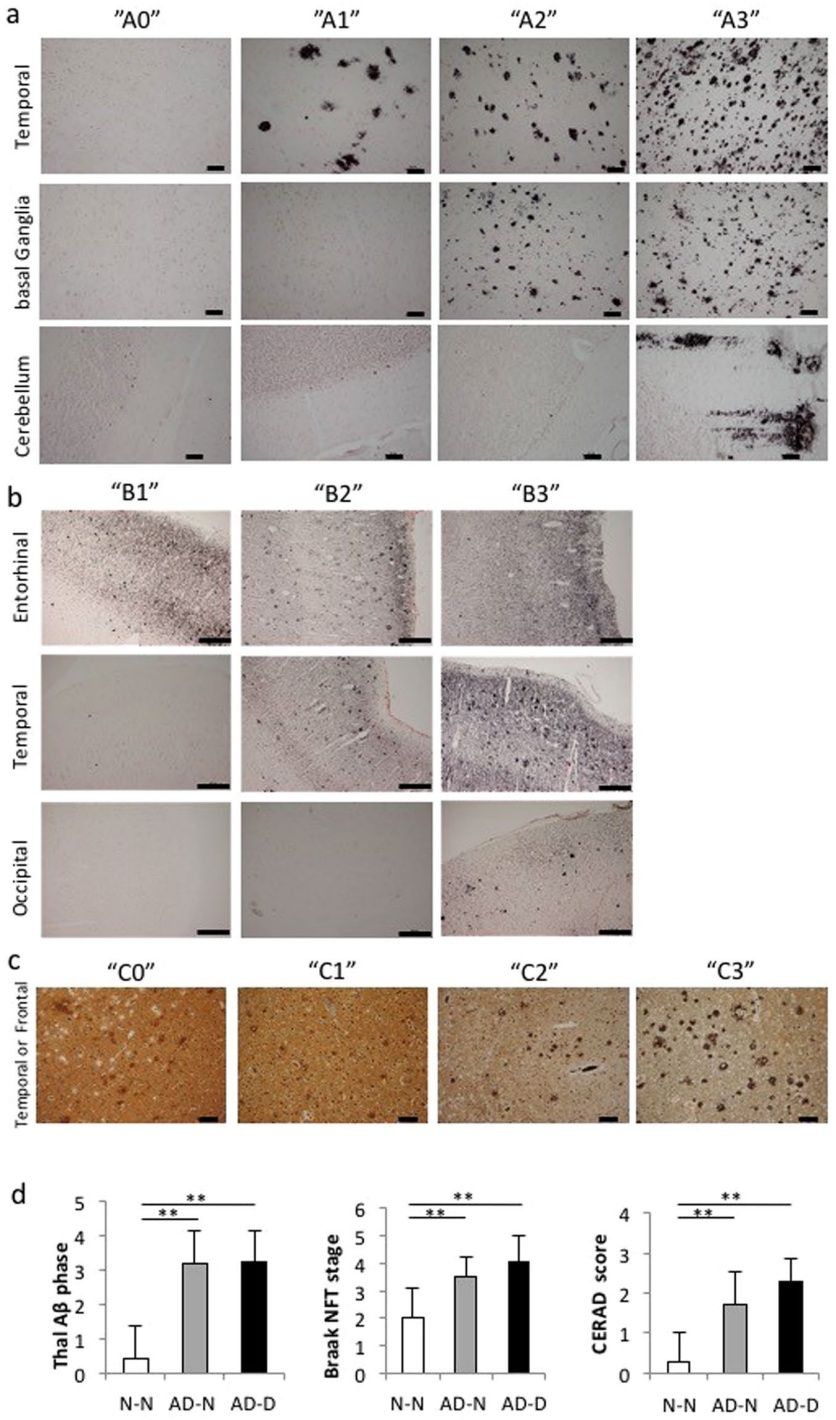
AD neuropathological diagnosis. Representative images of each score for the AD neuropathological changes. (**a**) The “A” score was determined after evaluation of the Thal Phase for Aβ plaques. The figure shows an example of “A0” in which no Aβ deposits are observed in any area, “A1” in which deposits are observed in the temporal area, “A2” in which deposits are observed in the temporal area and basal ganglia, and “A3” in which deposits are observed in the temporal area, basal ganglia and cerebellum (Bar, 100 µm). (**b**) The “B” score was determined after evaluation of the Braak NFT stage. The figure shows an example of “B1” in which the entorhinal region is PHF-tau positive, “B2” in which the entorhinal region and temporal area are PHF-tau positive, and “B3” in which the entorhinal region, and temporal and occipital areas are PHF-tau positive (Bar, 500 µm). (**c**) The “C” score was determined after evaluation of the CERAD neuritic plaque score in the neocortex area by Bielshowsky silver staining. The figure shows an example of “C0” with no neuritic plaques, “C1” with 1 to 5 plaques per 1 mm^2^, “C2” with 6 to 20 plaques per 1 mm^2^, and “C3” with over 20 plaques per 1 mm^2^ (Bar, 100 µm). (**d**) Each score of Thal Aβ phase, Braak NFT stage and CERAD neuritic plaque score is significantly higher in the AD-N and AD-D groups in comparison with the N-N group. However, no differences are observed between the AD-N and AD-D groups. **P < 0.01, Kruskal-Wallis test and Mann-Whitney U test. Values are means ± SD (N-N: n = 19, AD-N: n = 10, AD-D: n = 18).

After elimination of brains on the basis of the exclusion criteria, 51 brains were obtained for this study. We investigated age, years of education, Thal Aβ stage, Braak NFT stage, CERAD neuritic plaque score, and ABC score for each donor and brain (Table 1). For evaluation of neuropathological changes, the “A” and “B” in the “ABC score” were determined from the Aβ deposit- and NFT-affected regions, respectively, as described in Methods. Representative images of each A and B score are shown in Fig. 1a and b, respectively. “C” in the “ABC score” was evaluated from the density of plaques in the neocortex, as described in Methods. Representative images of each C score are shown in Fig. 1c. The overall AD pathological change was diagnosed from a combination of the three scores according to the NIA-AA Regan criteria. Based on these criteria, “Not” and “Low” were regarded as showing no AD pathological change, while “Intermediate” and “High” were regarded as showing AD neuropathological changes (Table 1).

**Table 1:** Subject demographics and AD neuropathological changes and cognitive function.

No.	age	sex	Education years	“A” Thal Aβ phase	“B” Braak stage	“C” CERAD score	ABC score	NIA-AA criteria	CDR	Group
1	71	M	20	0	I	None	A0 B1 C0	Not	0	N-N
2	74	M	12	0	I	None	A0 B1 C0	Not	0.5	N-N
3	86	F	8	0	II	None	A0 B1 C0	Not	0	N-N
4	94	F	—	0	I	None	A0 B1 C0	Not	0.5	N-N
5	73	M	—	0	I	None	A0 B1 C0	Not	0	N-N
6	77	M	12	0	IV	None	A0 B2 C0	Not	0	N-N
7	90	M	16	1	IV	None	A1 B2 C0	Low	0	N-N
8	90	M	12	2	II	Moderate	A1 B1 C2	Low	0.5	N-N
9	85	M	12	0	I	None	A0 B1 C0	Not	0.5	N-N
10	88	M	16	0	II	None	A0 B1 C0	Not	0	N-N
11	85	M	12	0	II	None	A0 B1 C0	Not	0	N-N
12	86	M	12	0	III	None	A0 B2 C0	Low	0.5	N-N
13	70	F	12	0	I	None	A0 B1 C0	Not	0	N-N
14	71	M	12	1	I	None	A1 B1 C0	Low	0	N-N
15	79	M	12	0	I	None	A0 B1 C0	Not	0	N-N
16	95	F	6	0	I	None	A0 B1 C0	Not	0	N-N
17	82	M	16	3	I	Moderate	A2 B1 C2	Low	0	N-N
18	80	F	—	0	I	None	A0 B1 C0	Low	0	N-N
19	81	F	16	0	I	None	A0 B1 C0	Not	0	N-N
20	101	F	—	3	IV	Sparse	A2 B2 C1	Intermediate	0	AD-N
21	82	M	12	5	III	None	A3 B2 C0	Intermediate	0.5	AD-N
22	88	F	6	4	IV	Moderate	A3 B2 C2	Intermediate	0	AD-N
23	99	F	8	3	III	Moderate	A2 B2 C2	Intermediate	0.5	AD-N
24	88	M	6	4	V	Frequent	A3 B3 C3	High	0	AD-N
25	77	F	12	2	III	Moderate	A1 B2 C2	Intermediate	0	AD-N
26	89	F	12	3	III	Moderate	A2 B2 C2	Intermediate	0	AD-N
27	98	M	6	3	III	Moderate	A2 B2 C2	Intermediate	0.5	AD-N
28	76	F	9	3	III	Moderate	A2 B2 C2	Intermediate	0	AD-N
29	81	M	16	2	IV	Moderate	A1 B2 C2	Intermediate	0	AD-N
30	92	F	6	5	IV	Frequent	A3 B2 C3	Intermediate	3	AD-D
31	71	F	10	5	IV	Moderate	A3 B2 C2	Intermediate	2	AD-D
32	95	F	10	5	V	Frequent	A3 B3 C3	High	3	AD-D
33	103	F	12	3	VI	Moderate	A2 B3 C2	Intermediate	2	AD-D
34	105	F	—	3	V	Moderate	A2 B3 C2	Intermediate	3	AD-D
35	90	M	8	5	V	Moderate	A3 B3 C2	Intermediate	3	AD-D
36	95	F	12	3	IV	Sparse	A2 B 2C1	Intermediate	3	AD-D
37	85	F	8	3	V	Frequent	A2 B3 C3	Intermediate	2	AD-D
38	91	F	9	3	IV	Frequent	A2 B 2C3	Intermediate	3	AD-D
39	100	F	6	3	III	Moderate	A2 B2 C2	Intermediate	3	AD-D
40	87	M	13	5	III	Moderate	A3 B2 C2	Intermediate	2	AD-D
41	88	M	—	2	III	Moderate	A1 B2 C2	Intermediate	3	AD-D
42	98	M	—	3	IV	Moderate	A2 B2 C2	Intermediate	2	AD-D
43	99	M	9	3	IV	Sparse	A2 B2 C1	Intermediate	3	AD-D
44	90	F	12	3	III	Sparse	A2 B2 C1	Intermediate	1	AD-D
45	76	F	—	3	IV	Moderate	A2 B2 C2	Intermediate	3	AD-D
46	90	M	9	3	III	Moderate	A2 B2 C2	Intermediate	3	AD-D
47	83	M	9	3	III	Moderate	A2 B2 C2	Intermediate	3	AD-D
	N-N:81.9±7.8, AD-N:87.9±9.1, AD-D:91±8.8	M: 24 F: 23	N-N:12.9±3.3, AD-N:9.7±3.5, AD-D:9.5±2.2			None: 16 Sparse 2: Moderate: 22 Frequent: 7		Not:13 Low: 4 Intermediate:28 High:2		N-N: 19 AD-N: 10 AD-D: 18

CDR = Clinical Dementia Rating scale; N-N = No AD neuropathological changes with no dementia.

AD-N = AD neuropathological changes with no dementia; AD-D = AD neuropathological changes with dementia.

Cognitive function was assessed by postmortem evaluation using the CDR scale. The questions on the CDR were answered by members of the bereaved family or other acquaintances of the donor (Supplementary Table 1). ‟No dementia” was determined as a CDR score of 0 or 0.5, and “Dementia” was determined as a CDR score of 1, 2 or 3 for convenience as previously reported^22–24^. Based on the AD neuropathological changes and CDR scores, the 51 brains were classified into 4 groups: (1) No AD neuropathological changes with No dementia; N-N, (2) No AD neuropathological changes with Dementia; N-D, (3) AD neuropathological changes with No dementia; AD-N, and (4) AD neuropathological changes with Dementia; AD-D. As the aim of this study was to reveal the neuropathological features that distinguish the AD-N group from the AD-D group, we excluded the N-D group (4 of 51 brains) as this group may have included other neurological deficits such as dementia with Lewy bodies or cerebrovascular dementia. We also evaluated the activities of daily living using the Katz Index and I-ADL score.

We found that 19 of 47 brains (41%) belonged to the N-N group, 10 of 47 (21%) to the AD-N group, and 18 of 47 (38%) to the AD-D group (Table 1). Average age at death was 81.9 ± 7.8 in the N-N group, 87.9 ± 9.1 in the AD-N group, and 91 ± 8.8 in the AD-D group (Table 1). Age at death was significantly higher in the AD-D group than in the N-N group (*P* = 0.006). However, no difference was found between the AD-N and AD-D groups. The causes of death in each group are shown in Supplementary Table 1. Years of education were significantly higher in the N-N group (12.9 ± 3.3) than in the AD-N (9.7 ± 3.5, *P* = 0.04) and AD-D groups (9.5 ± 2.2, *P* = 0.01) (Table 1).

Thal Aβ phase, Braak NFT stage, and CERAD neuritic plaque score were significantly higher in the AD-N and AD-D groups than in the N-N group (Fig. 1d). However, no differences were found between the AD-N and AD-D groups (Fig. 1d). Details of the percentage of each ABC score (A as Thal Aβ phase, B as Braak NFT stage, and C as CERAD neuritic plaque score) in demented and non-demented subjects are shown in Supplementary Table 3. As the entorhinal cortex (EC) is an important area for the evaluation of cognitive function, we investigated neuropathological changes in this area.

### The area of the EC

**Figure 2:**
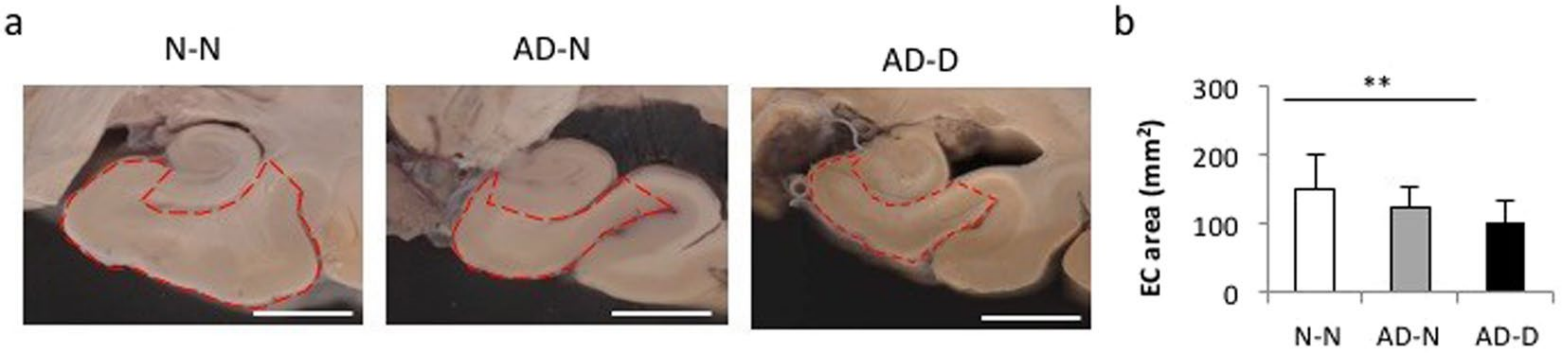
The area of the EC. (**a**) Scanned images of the coronal sections of the brain that cut through the lateral geniculate body and hippocampus are shown (Bar, 10 mm). (**b**) The area of the EC enclosed by red dotted lines is significantly lower in the AD-D group compared to that in the N-N group. No difference is observed between the AD-N and AD-D groups. **P < 0.01, one-way ANOVA, Tukey post-test. Values are means ± SD (N-N: n = 19, AD-N: n = 10, AD-D: n = 18).

Coronal sections of the brain that cut through the lateral geniculate body and hippocampus are shown in Fig. 2, and the area of the EC (enclosed by red dotted lines in Fig. 2a) was measured. The area was significantly decreased in the AD-D group compared to the N-N group (Fig. 2b). However, no difference was found between the AD-N and AD-D groups (Fig. 2b).

### Aβ and PHF-tau in the EC

**Figure 3:**
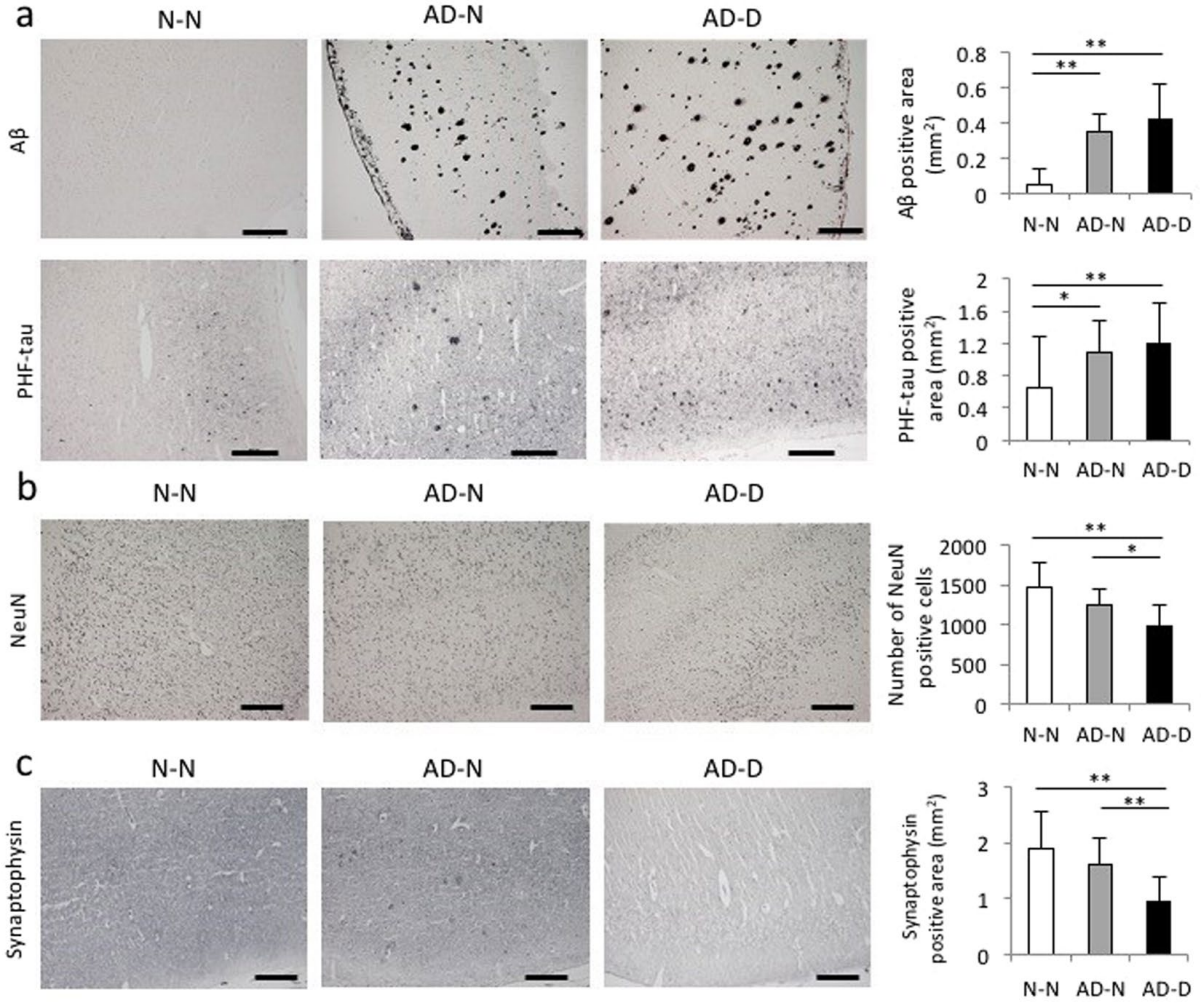
Immunohistochemical analysis of Aβ, PHF-tau, NeuN and synaptophysin in layer I-VI of the EC. (**a**) The Aβ- and PHF-tau-positive area is significantly higher in the AD-N and AD-D groups than in the N-N group. No difference is observed between the AD-N and AD-D groups (Bar, 500 µm). (**b**) The number of NeuN-positive cells in the EC is lower in the AD-D group compared to those in the N-N and AD-N groups. No difference is observed found in the number of NeuN-positive cells between the N-N and AD-N groups (Bar, 500 µm). (**c**) The synaptophysin-positive area is lower in the AD-D group than in the N-N and AD-N groups. No difference is observed between the N-N and AD-N groups (Bar, 500 µm). *P < 0.05, **P < 0.01, one-way ANOVA, Tukey post-test. Values are means ± SD (N-N: n = 19, AD-N: n = 10, AD-D: n = 18).

Aβ-positive area in the EC was significantly higher in the AD-N and AD-D groups than in the N-N group. Likewise, the PHF-tau-positive area in the EC was significantly higher in the AD-N and AD-D groups than in the N-N group (Fig. 3a). However, no differences were found in Aβ- and PHF-tau-positive areas between the AD-N and AD-D groups (Fig. 3a).

### The number of NeuN-positive cells and the synaptophysin-positive area in the EC

The number of NeuN-positive cells in the EC was lower in the AD-D group than in the N-N and AD-N groups (Fig. 3b). However, no difference was found in the number of NeuN-positive cells between the N-N and AD-N groups (Fig. 3b). The synaptophysin-positive area was lower in the AD-D group than in the N-N and AD-N groups, however no difference was found between the N-N and AD-N groups (Fig. 3c).

### The GFAP-positive area, the number of GFAP-positive astrocytes and astrocytic processes in layer I/II of the EC

**Figure 4:**
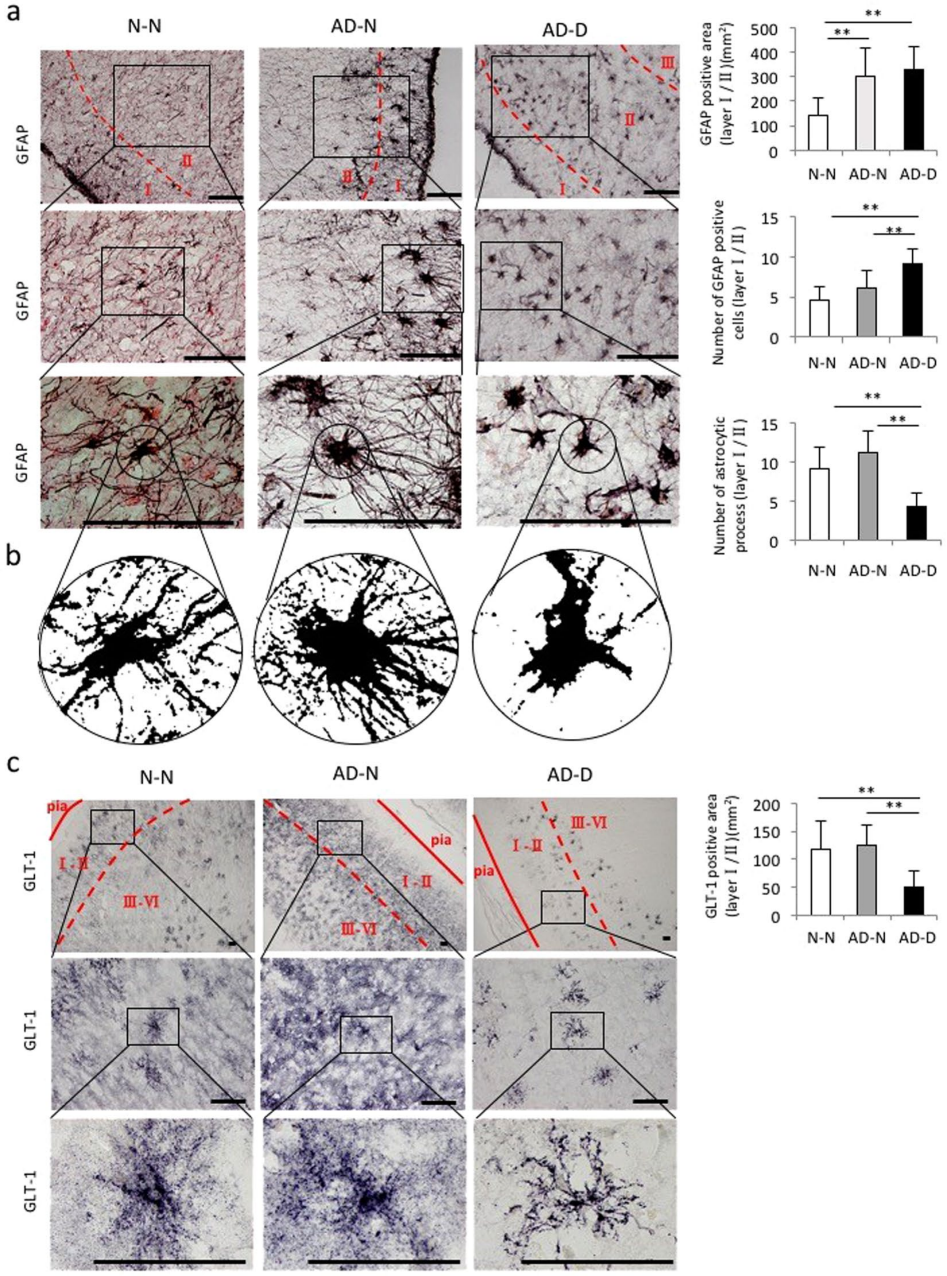
Immunohistochemical analysis of GFAP and GLT-1 in layer I/II of EC. (**a**) Images of GFAP-positive astrocytes shown at different magnifications (Bar = 100 µm). The GFAP-positive area is higher in the AD-N and AD-D groups than in the N-N group, while no difference is observed between the AD-N and AD-D groups. The number of GFAP-positive astrocytes is higher in AD-D group than in the N-N and AD-N groups, while no difference is observed between the N-N and AD-N groups. Astrocytic processes in the AD-N group appear extremely long, thick, and bushy, while those in the AD-D group appear short and thin. (**b**) The number of astrocytic processes observed 20 μm away from the soma is lower in the AD-D group than in the N-N and AD-N groups. (**c**) GLT-1 images are shown at different magnifications (Bar = 100 µm). The GLT-1-positive area is lower in the AD-D group than in the N-N and AD-N groups, while no difference is observed between the N-N and AD-N groups. The expression of GLT-1 is strong in both the proximal and distal astrocytic processes in the N-N and AD-N groups, while GLT-1 expression is weaker, especially in the distal astrocytic processes, in the AD-D group. **P < 0.01, one-way ANOVA, Tukey post-test. Values are means ± SD (N-N: n = 19, AD-N: n=10, AD-D: n = 18).

The GFAP-positive area in layer I/II of the EC was higher in the AD-N and AD-D groups than in the N-N group, however no difference was found between the AD-N and AD-D groups (Fig. 4a). The number of GFAP-positive astrocytes in layer I/II of the EC was higher in the AD-D group than in the N-N and AD-N groups (Fig. 4a). However, no difference was found in the number of GFAP-positive astrocytes between the N-N and AD-N groups (Fig. 4a). On the other hand, the number of GFAP-positive astrocytic processes was lower in the AD-D group compared to the N-N and AD-N groups (Fig. 4a). No difference was found in the number of GFAP-positive astrocytic processes between the N-N and AD-N groups (Fig. 4a). More characteristically, prominent morphological differences were observed in the astrocytic processes between the AD-N and AD-D groups. The GFAP-positive astrocytes in the AD-N group possessed long, thick, and bushy cytoplasmic processes, while those in the AD-D group possessed relatively short, thin cytoplasmic processes (Fig. 4b). In both the AD-N and AD-D groups, the perikarya was enlarged in comparison to that in the N-N group. In the N-N group, astrocytes were observed as an intermediate or mixed type between those in the AD-N and AD-D groups (Fig. 4b).

### The GFAP-positive area, the number of GFAP-positive astrocytes and astrocytic processes in layer III-VI of the EC

**Figure 5:**
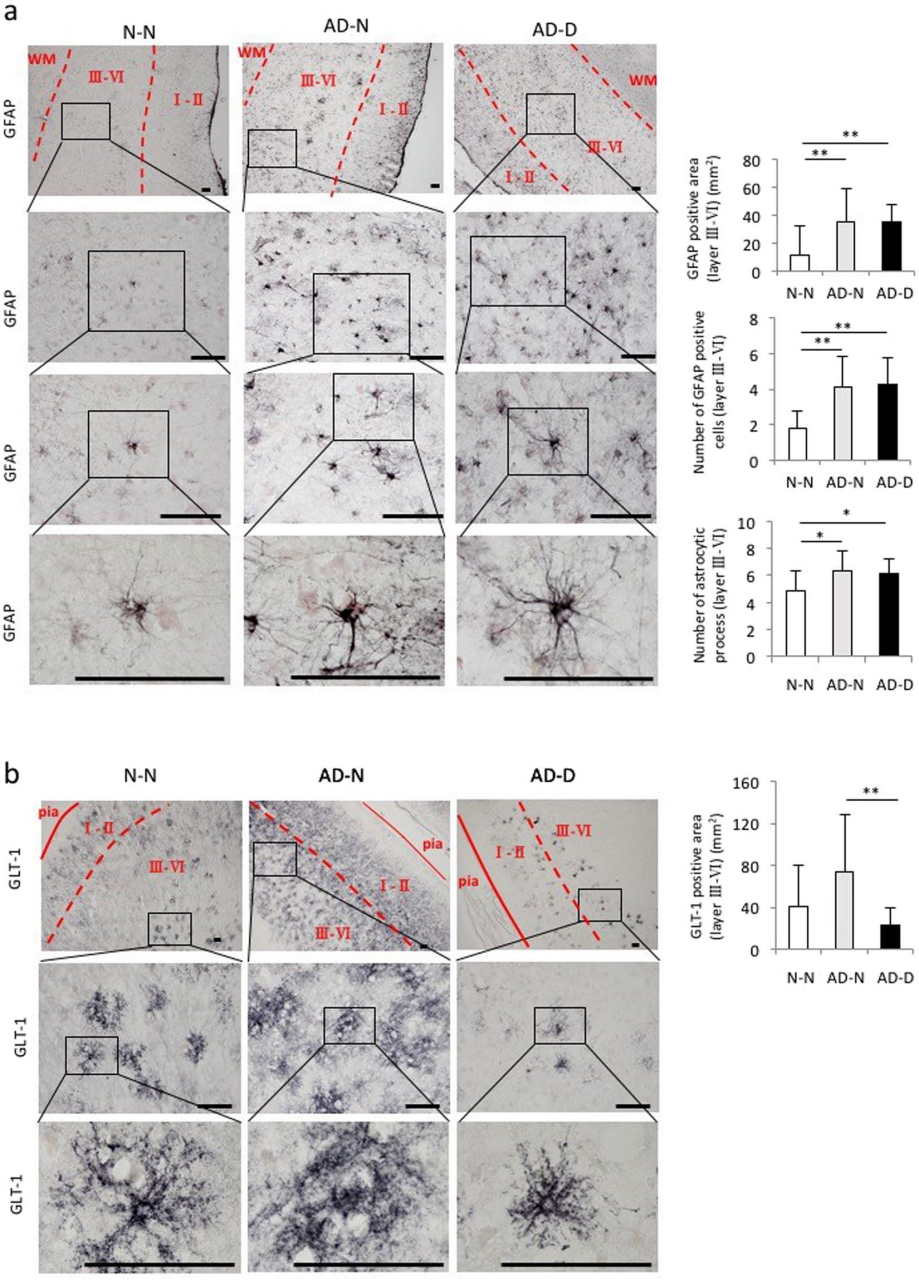
Immunohistochemical analysis of GFAP and GLT-1 in layer III-VI of EC. (**a**) Images of GFAP-positive astrocytes shown at different magnifications (Bar = 100 µm). The GFAP-positive area is higher in the AD-N and AD-D groups than in the N-N group, while no difference is observed between the AD-N and AD-D groups. The number of GFAP-positive astrocytes is higher in AD-N group and AD-D than in the N-N group, while no difference is observed between the AD-N and AD-D groups. The number of astrocytic processes observed 20 μm away from the soma is higher in the AD-N and AD-D groups than in the N-N group. (**b**) GLT-1 images are shown at different magnifications (Bar = 100 µm). The GLT-1-positive area is lower in the AD-D group than in the AD-N groups. The expression of GLT-1 is strong in both the proximal and distal astrocytic processes in the AD-N groups, while GLT-1 expression is weaker, especially in the distal astrocytic processes, in the AD-D group. *P < 0.05, **P < 0.01, one-way ANOVA, Tukey post-test. Values are means ± SD (N-N: n=19, AD-N: n = 10, AD-D: n = 18).

The GFAP-positive area in layer III-VI of the EC was higher in the AD-N and AD-D groups than in the N-N group, however no difference was found between the AD-N and AD-D groups (Fig. 5a). The number of GFAP-positive astrocytes and the number of astrocytic processes in layer III-VI of the EC was higher in both the AD-N and AD-D groups than in the N-N groups (Fig. 5a). However, no difference was found in the number of GFAP-positive astrocytes and astrocytic processes between the AD-N and AD-D groups (Fig. 5a). In both the AD-N and AD-D groups, GFAP-positive astrocytes showed enlarged perikarya and thick processes compared to that in the N-N group.

In contrast, no significant difference was found in the number of S100b-positive cells in layer III-VI of the EC, and no significant difference was found in the number of processes of S100b-positive cells among the three groups (Supplementary Figure 4a). Moreover, morphological differences in cell structures between the three groups were not found (Supplementary Figure 4a).

### The GLT-1-positive area and mRNA expression in the EC

The GLT-1-positive area in layer I/II of the EC was lower in the AD-D group than in the N-N and AD-N groups (Fig. 4c). However, no difference was found between the N-N and AD-N groups (Fig. 4c). The mRNA expression for GLT-1 in layer I/II of the EC was lower in the AD-D group than in the AD-N group (Supplementary Figure 3). However, no difference was found between the AD-D and N-N groups (Supplementary Figure 3).

The GLT-1-positive area in layer III-VI of the EC was lower in the AD-D group than in the AD-N group (Fig. 5b). However, no difference was found between the AD-D and N-N group (Fig. 5b). The percentage of GLT-1-positive cells in S100b-positive cells in layer III-VI of the EC was higher in the AD-N group than in the N-N and the AD-N groups (Supplementary Figure 4b). However, no difference was found between the AD-D and N-N groups (Supplementary Figure 4b). The GLT-1-positive area in each GLT-1/S100b-positive cell in layer III-VI of the EC was lower in the AD-D group than in the AD-N group (Supplementary Figure 4b). However, no difference was found between the AD-D and N-N groups (Supplementary Figure 4b).

The staining pattern of GLT-1-positive astrocytes varied among the three groups. In the N-N and AD-N groups, the expression of GLT-1 was strong in both the proximal and distal astrocytic processes in layer I/II and layer III-VI, while GLT-1 expression was weaker, especially in the distal astrocytic processes, in the AD-D group (Figs 4c, 5b and Supplementary Figure 4b).

### Factors correlated with CDR-SOB

Good correlations were found between the CDR-SOB and Katz index and IADL score as shown in Supplementary Figure 1. In addition, the GLT-1-positive area and the number of GFAP-positive astrocytic processes in the layer I/II showed good correlations with the CDR-SOB, while other factors, including age, area of the EC, Aβ-positive area, number of NeuN-positive cells, synaptophysin-positive area, the GLT-1-positive area and the number of GFAP-positive astrocytic processes in the layer III-VI were moderately or lower correlated with the CDR-SOB (Supplementary Figure 2).

## Discussion

To our knowledge, this is the first study to show that reactive astrocytes expressing GLT-1 in the EC are associated with the maintenance of cognitive function in spite of the progression of AD-specific pathological changes. Compared to the AD-D group, astrocytes in layer I/II of the EC from the AD-N group showed an increased number of astrocytic processes and longer or thicker forms with higher GLT-1 expression, while astrocytes in layer III-VI of the EC from the AD-N group showed higher GLT-1 expression. These activated astrocytes in the AD-N group may exert beneficial effects to protect neuronal transmission from the neurotoxicity associated with Aβ and NFT deposition.

We examined brains obtained from bodies aged over 70 years at death donated to Sapporo Medical University. The brain samples used covered various levels of cognitive function and were obtained from donors who died from a variety of causes. After careful exclusion of brains with non-AD neurological diseases such as cerebrovascular dementia or Parkinson’s disease-related dementia based on past medical history, we evaluated AD pathology based on the NIA-AA criteria “ABC score” composed of Thal Phase for Aβ plaques, Braak NFT stage and CERAD neuritic plaque score. These criteria are widely accepted as they reflect two important factors associated with AD pathology, Aβ and tau protein deposition^25^. Based on this report, “Not” and “Low” were evaluated as non-AD pathological change, while “Intermediate” and “High” were evaluated as AD pathological change.

To assess cognitive function, the CDR questionnaire was distributed to members of the bereaved families or other acquaintances. CDR is a common method of assessing cognitive function in the elderly, and can be used not only for diagnosis during life but also for postmortem diagnosis^26,27^. The validity and reliability of CDR for postmortem diagnosis were confirmed in a previous report^28^, and several clinico-pathological studies have applied this method^15,26^. We evaluated cognitive functions by questionnaires completed by family members but not by clinical physicians, as “No dementia” subjects are less likely to take cognitive tests given by clinical physicians when they were alive. In the present study, the cognitive level at 6 months before death was evaluated with CDR 0 (absent) and CDR 0.5 (questionable) classified as “No dementia”, and CDR 1, 2, and 3 classified as “Dementia,” as previously reported^22–24^. We also evaluated the activities of daily living using the Katz index and I-ADL, and both scores showed a good correlation with CDR, as reported previously^27,29,30^. Moreover, we confirmed the good inter-rater reliability of CDR between two the raters (EK and MN). Thus, the results of CDR were considered reliable.

In this study, the percentage of non-demented subjects with AD pathology was larger than that in previous studies. With regard to the Braak stage, 48% of the subjects with stage III + IV(12 of 25 cases) and 17% of the subjects with stage V + VI (1 of 6 cases) showed no dementia in this study (Supplementary Table 3). On the other hand, 32% of the subjects with stage III + IV and 8% of the subjects with stage V + VI showed no dementia in the Nun study^11^. With regard to the CERAD score, 40% of the subjects with moderate or frequent neuritic plaques (C2 or 3) showed no dementia in this study (10 of 25 cases) (Supplementary Table 3), while 34.7% of the subjects with C2 or 3 showed no dementia in the MRC CFAS study^10^. The discrepancies between the present and previous studies regarding the percentages of subjects with no dementia but with AD pathology might be due to the fact that 57% of the subjects (27 of 47 cases) lived at home during the final 6 months before death and had frequent contact with their family members (Supplementary Table 1, Supplementary Table 2), as a lack social interaction is closely related to the onset of dementia in the elderly^30^.

In our study, there were no differences in age or length of education between the AD-N and AD-D groups, and no differences were found in Thal Aβ phase, Braak NFT stage or CERAD score between the two groups. These results indicate that the cognitive impairment in the AD-D group might not be explained by aging, length of education, or deposition of Aβ plaques or tau accumulation in the brain. Therefore, we examined factors other than Aβ plaques and tau accumulation that may cause cognitive impairment in subjects with AD pathology.

The results revealed no differences in the accumulation of Aβ or PHF-tau in the EC between the AD-N and AD-D groups; however, the number of NeuN-positive cells and the area positive for synaptophysin were higher in the AD-N group than in the AD-D group. These results were in agreement with the findings of previous studies in which preserved neuronal number and synaptic density were shown in non-demented subjects with AD pathology^15,16^. As for the hippocampal volume, previous studies have shown that the hippocampal volume is correlated with the neuronal number^31^, and a reduction in volume, as assessed by MRI, is used for the diagnosis of AD^32^. Our results showed that the two-dimensional area of the EC was unchanged between the AD-N and AD-D groups, suggesting that the two-dimensional cross-section of the EC, at least, is not correlated with cognitive function.

The most characteristic findings were the differences in the morphology of the astrocytes in layer I/II of the EC between the AD-N and AD-D groups, although both groups exhibited reactive astrocytes with a larger GFAP-positive area than did the N-N group. Astrocytes in the AD-N group possessed longer or thicker processes while those in the AD-D group possessed shorter or thinner processes. Astrocytes located in layers I/II are known as “interlaminar astrocytes” and are characterized by the presence of millimeter-long projections terminating in layer III to IV^33^. It has been reported that in AD patients, disruption of the processes of these interlaminar astrocytes is correlated with dysfunction in neuronal transmission support^34^. In the present study, the longer or thicker astrocytic processes found in layer I/II extended to layer III to IV, where the astrocytic processes may play important roles in the synaptic transmission of neurons connected to the CA1 region^35,36^.

In contrast to astrocytes in layer I/II, there were no differences in the morphology of the astrocytes observed in layer III-VI between the AD-N and AD-D groups, although both groups exhibited a larger GFAP-positive area than did the N-N group. Astrocytes in layer III-VI, known as “protoplasmic astrocytes,” are spherical and possess several main processes which are accompanied by very thin branches^17^. Therefore, it remains possible that GFAP-positive reactions do not always detect the whole branches of very thin astrocytic processes in layer III-VI. In contrast to GFAP positive astrocytes, there were no differences in both the number of cells and processes in S100b positive astrocytes in layer III-VI. The subpopulation of astrocytes has been reported to be positive for S100b but negative for GFAP^37^, and S100b-positive reaction is localized around the perikarya of astrocytes^38^. Therefore, GFAP staining seems to be more suitable for detecting the reactive astrocytes which possess processes than S100b staining.

Reactive astrocytes have also been shown to have diphasic functions^39^, including both beneficial effects, such as the formation of a barrier to confine lesions, promotion of blood-brain barrier repair, inhibition of synaptic loss and slowing of neurodegenerative disease progression, and harmful effects, such as the production of reactive oxygen species, secretion of cytokines and inhibition of synapse and axon regeneration^40^. In previous reports, the expression of inflammatory cytokines, such as IL-1β, was higher in demented patients with AD pathology, compared to the non-demented patients with AD pathology^41^. Moreover, a single astrocyte in the human brain is associated with around 2 million synapses via its processes, which support memory formation^42^. Therefore, a diminished number of astrocytic processes leads to reduced glutamate transport and insufficient communication across gap junctions^43^.

To clarify the beneficial function of reactive astrocytes in the AD-N group, we examined the expression of GLT-1, which is a major glutamate transporter that uptakes excess glutamate to prevent neuronal excitotoxicity^44,45^, in the astrocytes in each group. The GLT-1 expression was detected by immunohistochemistry and its validity was confirmed by a quantitative RT-PCR. We observed a higher level of GLT-1 expression in astrocytes in the AD-N group compared to that in the AD-D group in both layer I/II and layer III-VI, suggesting that astrocytes in the AD-N group play beneficial roles in neuronal functions. The discrepancies in GLT-1 expression and morphology between astrocytes in the AD-N and AD-D groups in layer III-VI can be explained by the fact that astrocytes in the AD-N groups express high levels of GLT-1 in the very thin processes, but they were not detected completely by GFAP immunohistochemistry^46^. The higher expression of GLT-1 in astrocytes in layer III-VI from AD-N group was also confirmed by the overlapping staining of GLT-1 and S100b. In fact, the percentages of GLT-1-positive cells in S100b-positive cells and GLT-1 expression in each cell were higher in the AD-N group than in the N-N and the AD-N groups. Previous studies have shown that a reduction in GLT-1 is related with the cognitive impairment in AD^47,48^, and functional abnormalities in GLT-1 induce synaptic loss^49^. In the present study, reactive astrocytes in the AD-D group showed weak GLT-1 expression in spite of a higher number of cells, suggesting that these reactive astrocytes may not function adequately in the brain.

Various factors are known to influence the number of astrocytic processes as well as GLT-1 expression; for example, the ramification of astrocytic processes is increased by physical exercise or an enriched environment^50,51^, and GLT-1 expression is up-regulated by treadmill training^52^. Furthermore, GLT-1 expression and the number of astrocytic processes are increased through the memory formation processes^53^. Although the details of daily life and intellectual activity of the donors in the present study were not known, the potential for reactive astrocytes to be converted from non-activated to activated type and the maintenance of GLT-1 expression might be key factors in the preservation of normal cognitive function, even in the presence of AD pathology.

In conclusion, activated forms of astrocytes with higher GLT-1 expression are associated with cognitive normal subjects with the AD pathology in the brain. Identification of methods to convert reactive astrocytes into this beneficial type appears to afford a promising approach to the prevention of AD.

## Methods

### Subjects

All brains were obtained from the body donation program run by the Sapporo Medical University (Shiragiku-kai). This program consists of members aged 70 years or older who donate their bodies for medical education and research after death. The donation was agreed to by the members while they are alive. There were no prisoners in the subjects of this study. The protocols were approved by the Sapporo Medical University Ethics Committee and informed consent was obtained from all participants. All study methods were performed in accordance with the relevant guidelines and regulations of Sapporo Medical University.

Cognitive function was assessed by postmortem evaluation of informant questionnaires as reported previously^28^. The questionnaires consisted of the Japanese version of the Clinical Dementia Rating Scale (CDR)^22,24,54^, Katz Index^55^ and I-ADL^56^. Details of CDR are described in Supplementary information.

### AD neuropathological diagnosis

For evaluation of AD neuropathological changes, we applied three assessment scales: the Thal Phase for Aβ plaques^57^, Braak NFT stage^58,59^ and CERAD neuritic plaque score^60^. Details of evaluation of each score are described in Supplementary information.

After assessing each score of the “ABC”, we classified each brain as “Not”, “Low”, “Intermediate” or “High” based on a combination of the three scores according to guidelines of National Institute on Aging-Alzheimer’s Association^25^. “Not” and “Low” corresponded to no AD neuropathological change, while “Intermediate” and “High” corresponded to AD neuropathological change. These procedures were applied to each brain with the assessors blinded to the donor.

### Immunohistochemistry

Whole brain samples were fixed in 10% formalin. Blocks of the target area were cut, and then immersed in 15% sucrose solutions. For immunohistochemical analysis, coronal sections were cut into 20 μm thick frozen sections and obtained every 100 μm. A standard avidin-biotin complex (ABC) method was used for the analysis. Details of antibodies are described in Supplementary information.

### Statistical analysis

One-way ANOVA followed by Tukey post hoc comparison was used to detect differences among the three groups for variables with normal distributions. Non-parametric Kruskal-Wallis test and Mann-Whitney U test were used to detect differences among groups for variables with non-normal distributions. Bartlett’s test was used to confirm the data distributions. Spearmann’s correlation coefficient was used to confirm correlations between the CDR-SOB and Katz Index, I-ADL, age, area of the EC, Aβ-positive area, PHF-tau-positive area, number of NeuN-positive cells and synaptophysin-positive area. Statistical significance was set at 5% and data are presented as mean ± standard deviation (SD). All statistical analyses were performed using R software (version 3.3.2).

## Electronic supplementary material

Supplementary information
