# Supplementary material for: Activated forms of astrocytes with higher GLT-1 expression are associated with cognitive normal subjects with Alzheimer pathology in human brain
Source: Sci Rep. 2018 Jan 26;8:1712. doi: 10.1038/s41598-018-19442-7 (PMC5786045; doi:10.1038/s41598-018-19442-7)
Supplement: Supplementary file 1 — Supplementary information [file 41598_2018_19442_MOESM1_ESM.pdf]

Supplementary information

1. Supplementary Tables
2. Supplementary Figures
3. Methods
4. References

Activated forms of astrocytes with higher GLT-1 expression are associated with cognitive normal subjects with Alzheimer pathology in human brain

Eiji Kobayashi, Masako Nakano, Kenta Kubota, Nobuaki Himuro, Shougo Mizoguchi, Takako Chikenji, Miho Otani, Yuka Mizue, Kanna Nagaishi, Mineko Fujimiya

Supplementary Table 1. Characteristics of subjects

	N-N (n=19)	AD-N (n=10)	AD-D (n=18)	Total (n=47)
Cause of death, n (%)				
Heart failure, vascular disease	7	3	3	13 (28%)
Carcinoma	6	2	-	8 (17%)
Senility death	1	1	6	8 (17%)
Pneumonia	4	-	2	6 (13%)
Septicemia	-	2	2	4 (9%)
Digestive disease	1	1	1	3 (6%)
Kidney failure	-	1	-	1 (2%)
other	-	-	1	1 (2%)
Living location*, n (%)				
Home	16	8	3	27 (57%)
Nursing home	2	1	13	16 (34%)
Hospital	1	1	2	4 (9%)

* Information at 6month before death.

Supplementary Table 2. Characteristics of informants

	N-N (n=19)	AD-N (n=10)	AD-D (n=18)	Total (n=47)
Age	68±12.6	65.5±17.7	67.5±15.9	67±12.1
Sex, n				
Male	1	2	8	11(23%)
Female	18	8	10	36 (77%)
Relations with subjects, n (%)				
child, grandchild	13	5	6	24 (49%)
spouse	13	3	3	19 (40%)
nephew, niece	-	2	5	7 (15%)
brother, sister	1	-	4	5 (11%)
others	2		-	2 (4%)
Contact with subjects*, n (%)				
everyday	13	5	6	24 (49%)
3~4days/week	1	1	1	3 (6%)
1~2days/week	2	1	1	4 (9%)
less than one day/week	3	3	10	16 (34%)
post mortem interval, days	447.7±219	419.8±199	421.5±212.4	429.7±210.1

* Information at 6month before death.

Supplementary Table 3. Number of subjects classified in each criteria of AD neuropathological diagnosis by dementia status.

**AD neuropathological diagnosis**	**No Dementia (n=29)**	**Dementia (n=18)**
"A" Thal Aβ phase		
0	15 (34%)	0
1	2 (7%)	0
2	3 (10%)	1 (5%)
3	6 (21%)	12 (67%)
4	2 (7%)	0
5	1 (3%)	5 (28%)
"B" Braak NFT stage		
Ⅰ	12 (41%)	0
Ⅱ	4 (14%)	0
Ⅲ	7 (24%)	6 (33%)
Ⅳ	5 (17%)	7 (39%)
Ⅴ	1 (3%)	4 (22%)
Ⅵ	0	1 (6%)
"C" CERAD neuritic plaque score		
None	18 (62%)	0
Sparse	1 (4%)	3 (17%)
Moderate	9 (30%)	11 (61%)
Frequent	1 (4%)	4 (22%)

Supplementary Figure 1.

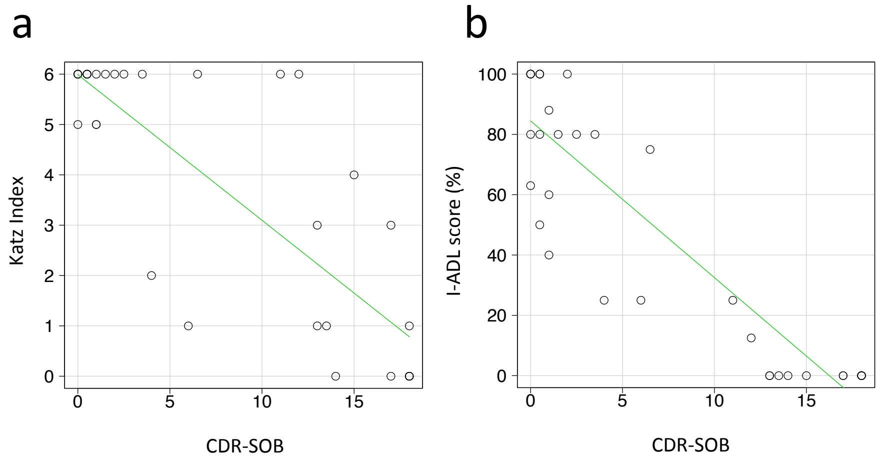

Supplementary Figure 1. Factors correlated with CDR-SOB. Factors well correlated with CDR-SOB are (a) Katz Index (rs = -0.73, $P < 0.001$) and (b) I-ADL score (rs = -0.79, $P < 0.001$). (rs: Spearmann's rank correlation coefficient).

Supplementary Figure 2.

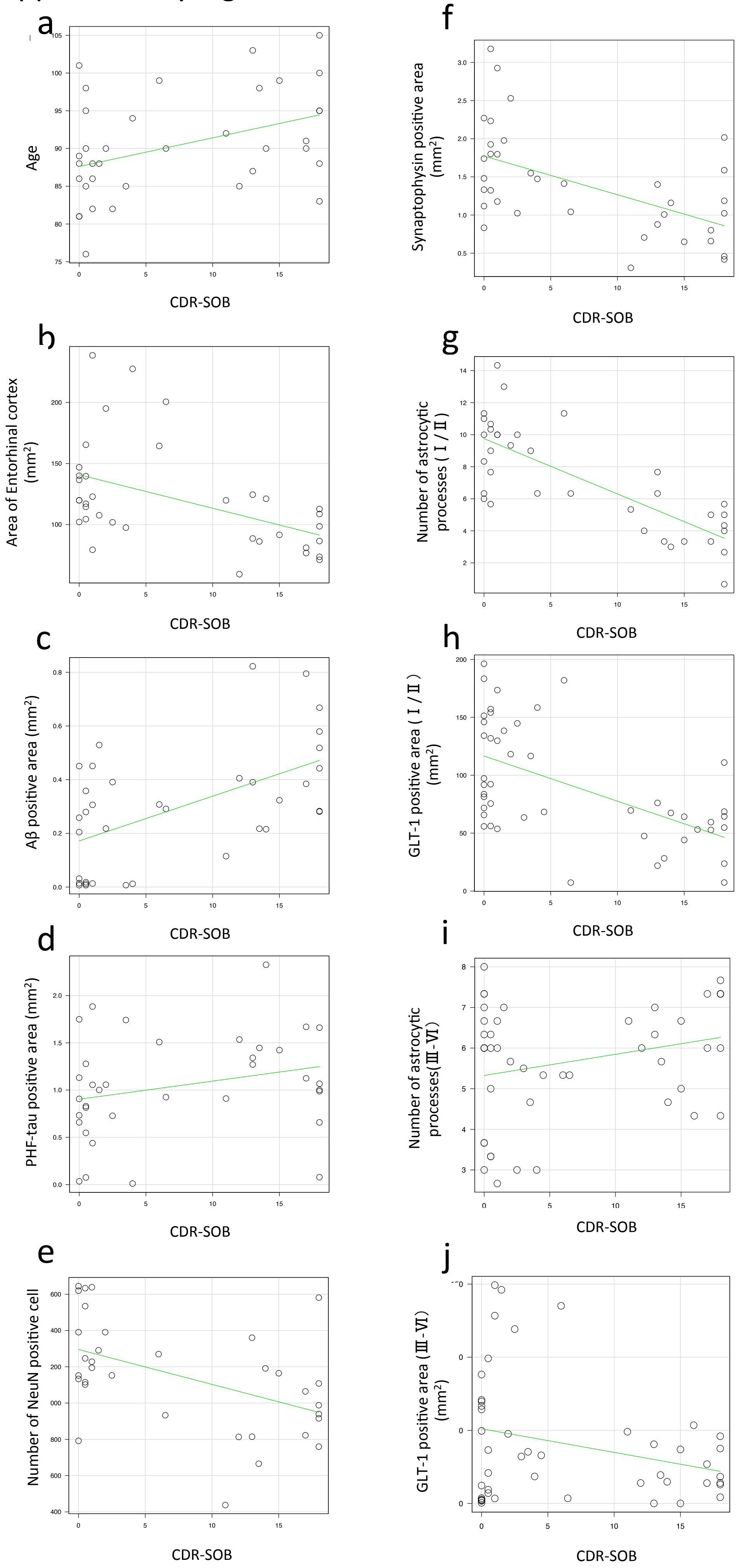

Supplementary Figure 2. Correlations between other factors and CDR-SOB. The factors correlated with CDR-SOB include (a) age (rs = -0.4, $P < 0.05$), (b) area of the EC (rs = -0.51, $P < 0.01$), (c) Aβ-positive area (rs = 0.51, $P < 0.01$), (d) PHF-tau-positive area (rs = 0.21, n.s), (e) number of NeuN-positive cells (rs = -0.44, P < 0.01), (f) synaptophysin positive area (rs = 0.48, $P < 0.01$), (g) number of astrocytic processes (I/II) (rs = -0.71, $P < 0.001$), and (h) GLT-1-positive area (I/II) (rs = -0.57, $P < 0.001$,(i) number of astrocytic processes (III-VI) (rs = -0.09, n.s), and (h) GLT-1-positive area (III-VI) (rs = -0.09, n.s). (rs: Spearmann's rank correlation coefficient).

Supplementary Figure 3.

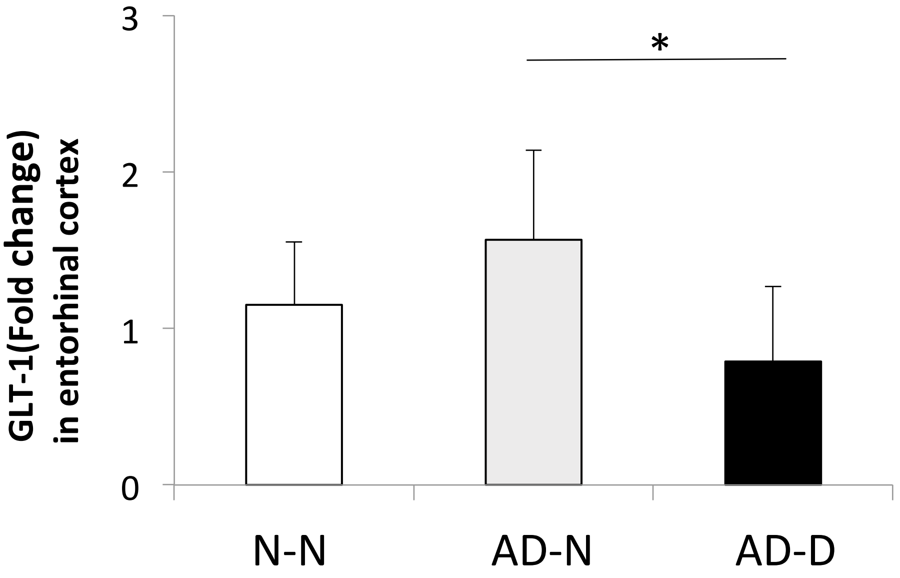

Supplementary Figure 3. The mRNA expression for GLT-1 in layer I and II of the entorhinal cortex (EC) was lower in the AD-D group than in the AD-N group. No difference was found between the AD-D and N-N groups. *P < 0.05, one-way ANOVA, Tukey post-test. Values are means ± SD (N-N: n=6, AD-N: n=6, AD-D: n=6).

Supplementary Figure 4.

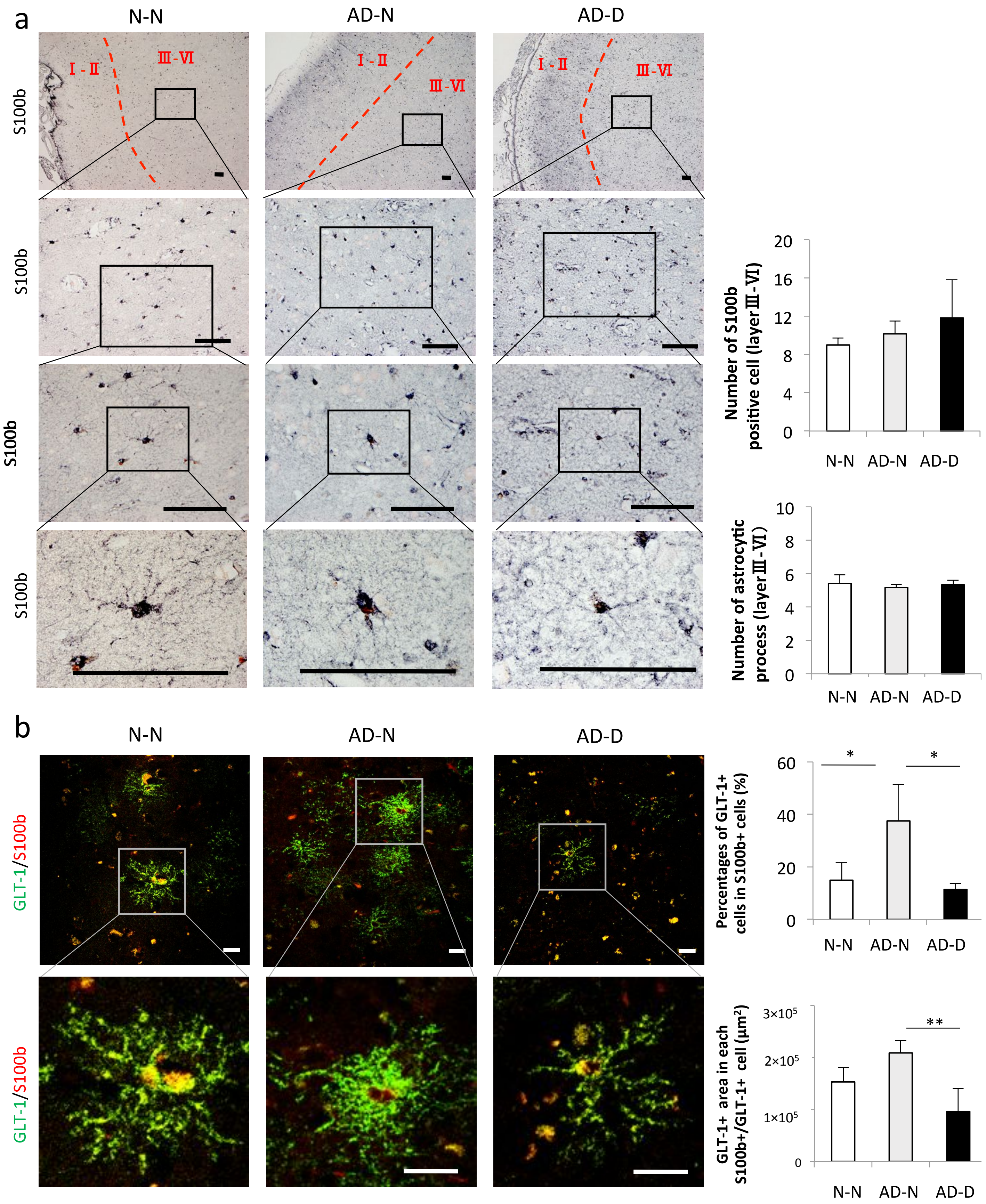

Supplementary Figure 4. Immunohistochemical analysis of S100b and GLT-1 in layer III-VI of EC. (**a**) Images of S100b-positive cells shown at different magnifications (Bar = 100 μm). No significant difference is found in the number of S100b-positive cells and the number of cell processes observed 20 μm away from the soma among the three groups. Values are means ± SD (N-N: n=19, AD-N: n=10, AD-D: n=18). (**b**) Overlap staining with GLT-1 and S100b images are shown (Bar = 20 μm). The percentages of GLT-1-positive cells in S100b-positive cells in the layer III-VI of the EC is higher in the AD-N group than in the N-N and the AD-N groups, while no difference is found between the AD-D and N-N groups. The GLT-1-positive area in each GLT-1/S100b-positive cells, which are stained with S100b in perikarya and stained with GLT-1 in the distal cell processes, is lower in the AD-D group than in the AD-N group, while no difference is found between the AD-D and N-N groups. *P <0.05, ** P < 0.01, one-way ANOVA, Tukey post-test. Values are means ± SD (N-N: n=4, AD-N: n=4, AD-D: n=4).

Methods.

Exclusion criteria.

To exclude neurological disease other than AD as far as possible, we set the following exclusion criteria, 1) brains with macroscopic infarction or hemorrhage (> 10mm), 2) subjects with a past history of cerebrovascular disease, mental disorders or neurological deficits except AD (e.g., depression, Lewy body disease), and 3) subjects for whom a CDR score was difficult to ascertain (e.g., a lack of information).

Questionnaires.

Questionnaires were completed by bereaved family members or other acquaintances of the deceased (Supplementary table 2). Where items in the questionnaires were unclear, an additional interview was carried out by telephone. The score of CDR show the diverse of cognitive function as absent, questionable, mild, moderate, or severe (CDR 0, 0.5, 1, 2, 3 respectively)[1-3]. In present study, "No dementia" was determined as a CDR score of 0 or 0.5, and "Dementia" was determined as a CDR score of 1, 2 or 3 for convenience as previously reported[1,2,4]. The CDR score was assessed by two evaluators (E.K and M.N) independently, and the inter-rater reliability of this test was very high (Kendall's rank correlation tau = 0.9, $P < 0.001$). In addition, the CDR Sum of Boxes (CDR-SOB) was calculated as reported previously[5]. All informants were asked to provide information on the donors at 6 months prior to death. Additional information including past medical history, years of education and address at 6 months before death was obtained from the questionnaires. The cause of death was also confirmed from the Certificate of Death in each case (Supplementary Table 1).

AD neuropathological diagnosis.

Brains were removed and fixed in 10% formalin. The left hemispheres were removed and cut into blocks to provide 5 to 10mm coronal sections. We obtained sections for 8 areas of each brain: (1) hippocampus and entorhinal cortex, (2) middle frontal gyrus, (3) superior and middle temporal gyrus, (4) inferior parietal lobule, (5) occipital cortex (BA 17 and 18), (6) midbrain (including the Substantia nigra), (7) cerebellum cortex and dentate nucleus, and (8) basal ganglia with basal nucleus of Mynert, based on previous reports[6].

For evaluation of Thal Phase, Aβ immunohistochemical analysis was performed for each area. Phase 0 denotes the absence of Aβ deposits in any area; phase 1 was characterized by deposits exclusively in the neocortex; phase 2 by additional involvement of the allocortex including CA1 and the entorhinal region; phase 3 by further deposits in the subcortical region including the basal ganglia, and phase 4 and 5 by additional deposits in the midbrain and cerebellum, respectively. After Thal Phase evaluation, we assessed the "A" in the "ABC score," which is limited to four stages (A0 corresponds to Phase 0, A1 includes Phase 1 and 2, A2 corresponds to Phase 3, and A3 includes Phase 4 and 5).

For evaluation of the Braak stage, we undertook phospho-tau immunohistochemical analysis for each area[6]. Stage I was characterized by NFTs in the transentorhinal region of the hippocampus; stage II by extended NFTs in the entorhinal cortex region of the hippocampus; stage III by further extension in the fusiform in the temporal cortex and lingual gyri in the occipital cortex; stage IV by increased NFT density in the sites affected in stage III; stage V by extension of the neocortical NFTs into the frontal and superior temporal gyrus; and stage VI by severe involvement of most areas of the neocortex and extension into the striate area. After evaluation of the Braak stage, we assessed the "B" in the "ABC score," which is again limited to four stages (B0 corresponds to None, B1 includes Stage I and II, B2 includes Stage III and IV, and B3 includes Stage V and VI).

For evaluation of the CERAD score, we performed Bielschowsky staining of the neocortical area as described previously[6,7]. No neuritic plaques was scored as "None;" an increasing density of plaques (1 to 5 plaques per 1 mm^2) was scored as "Sparse;" and an even greater plaque density was determined as "Moderate" or "Frequent" (6 to 20 plaques or over 20 plaques per 1 mm^2, respectively). After evaluation of the CERAD score, we assessed the "C" in the "ABC score," which consists of four stages (C0 corresponds to "None", C1 to "Sparse", C2 to "Moderate", and C3 to "Frequent").

Macroscopic assessment of the EC.

We scanned the coronal sections of the left hemisphere that include the lateral geniculate body and hippocampus by digital scanner. The area of the EC was then evaluated by Image J as reported previously[8].

Immunohistochemistry.

The sections were incubated for 2 days at 4 ^oC with primary antibodies against beta-amyloid (D54D2) (rabbit mAb, 1:100; Cell signaling, Danvers, MA, USA), PHF-tau (Anti-Human Monoclonal, 1:80; Thermo Fisher Scientific, Waltham, MA, USA), NeuN (rabbit polyclonal, 1:250; Millipore, Darmstadt, Germany), synaptophysin (rabbit polyclonal, 1:250; Sigma-Aldrich, St. Louis, MO, USA), GFAP (chicken polyclonal, 1:500; Millipore), S100b (rabbit polyclonal, 1:200; abcam, Cambridge, UK), and GLT-1 (mouse GLT-1, 1:100; FRONTIER INSTITUTE, Hokkaido, Japan). After blocking with 0.1% H_2O_2, sections were incubated with biotinylated secondary antibodies, anti-rabbit IgG (1:1000; Jackson ImmunoResearch, West Grove, PA, USA), anti-mouse IgG (1:1000; Jackson ImmunoResearch) and anti-chiken IgY (1:1000; Jackson ImmunoResearch), for 2h at room temperature. Sections were incubated in AB complex for 1.5h at room temperature, and then DAB solution mixed with 1% H_2O_2 and Nickel Ammonium Sulfate was added to each section, which were then further incubated for 60 min at room temperature. Finally, nuclei were stained with neutral red.

Quantitative analysis of DAB staining.

Quantitative analysis was performed on the EC. The area positive for Aβ and PHF-tau was evaluated in 6 different fields in layer I -VI per brain (3 fields of 3.2 × 2.4 mm per section). The number of NeuN-positive cells and the area of synaptophysin were evaluated in 6 different fields in layer of I -VI per brain (3 fields of 3.2 × 2.4 mm per section). The area positive for GFAP was evaluated in 6 different fields in layer I/II and layer III-VI each, per brain (3 fields of 1280 × 960 μm per section). The number of GFAP-positive cells were counted in 6 different fields in layer I/II and layer III-VI each, per brain (3 fields of 150×150 μm per section). The number of the astrocytic processes observed 20 μm away from the soma was counted in 12 different cells in layer I/II and layer III-VI each, per brain (6 cells per section) by Sholl analysis[9]. The number of S100b-positive cells were counted in 6 different fields in layer III-VI each, per brain (3 fields of 150×150 μm per section). The number of the S100b-positive cell processes observed 20 μm away from the soma was counted in 12 different cells in layer III-VI each, per brain (6 cells per section) by Sholl analysis[9]. The area positive for GLT-1 was also evaluated in 6 different fields in layer I/II and layer III-VI each, per brain (3 fields of 640 × 480 μm per section).

All sections were assessed by light microscopy (Nikon Eclipse), and NIS-Elements software 3.22.00 (Nikon) was used to obtain images. All immunohistochemical analyses were performed using Image J 1.5 (National Institute of Health) after binarization.

Immunofluorescences staining.

For immunofluorescence staining analysis, coronal sections were cut into 5μm thick paraffin-embedded sections and obtained every 25μm. The sections were incubated for 2 days at 4℃ with primary antibodies against S100b (rabbit polyclonal, 1:200; abcam) and GLT-1 (guinea pig polyclonal, 1:100; FRONTIER INSTITUTE). For secondary antibodies, Cy3-labeled anti-rabbit IgG (Jackson ImmunoResearch,) and Alexa Fluor 488-labeled anti-guinea pig IgG (Millipore) diluted in 1:500 were used. Sections were observed under confocal laser scanning microscopy (Nikon A1, Tokyo, Japan).

The percentage of GLT-1-positive cells in S100b-positive cells were evaluated in the layer III-VI of EC. The GLT-1-positive area in each GLT-1/ S100b-poitive cells were evaluated in 4 different cells in layer III to VI each, per brain. The GLT-1/ S100b- positive cells were defined as the cells which were stained with S100b in perikarya and stained with GLT-1 in the distal cell processes.

Total RNA isolation, Reverse transcription (RT) and real time RT-PCR

Total RNA was isolated from the area of layer I/II in entorhinal cortex which is paraffin-embedded sections by using the Invitrogen Recover All™ Total Nucleic Acid Isolation kit (Thermo Fisher Scientific). The complementary DNA (cDNA) was synthesized by using a Sensiscript Reverse Transcriptase (RT) Kit (QIAGEN, Hilden, Germany), and mRNA levels were measured by RT-PCR using Power SYBR Green PCR Master Mix (Thermo Fisher Scientific). GLT-1 mRNA was detected by primers: GGGCTTCTTCGCTTGGCATCTC (forward) and CTCCGGCACCTCAGTCACAGTC (reverse). Glyceraldehyde-3-phosphate dehydrogenase (GAPDH) mRNA was detected as an internal standard with primers: ATTGCCCTCAACGACCACTT (forward) and TGCTGTAGCCAAATTCGTTGTC (reverse). The relative changes in gene expression was determined by the $2^{-\Delta\Delta Ct}$ method.

References

1. Hughes, C. P., Berg, L., Danziger, W. L., Coben, L. A. & Martin, R. L. A new clinical scale for the staging of dementia. Br J Psychiatry 140, 566-572 (1982).
2. Morris, J. C. The Clinical Dementia Rating (CDR): current version and scoring rules. Neurology 43, 2412-2414 (1993).
3. Sugishita, M. & Furukawa, K. [Clinical Dementia Rating (CDR)]. Nihon Rinsho 69 Suppl 8, 413-417 (2011).
4. Jellinger, K. A. Clinicopathological analysis of dementia disorders in the elderly--an update. J Alzheimers Dis 9, 61-70 (2006).
5. O'Bryant, S. E. et al. Staging dementia using Clinical Dementia Rating Scale Sum of Boxes scores: a Texas Alzheimer's research consortium study. Arch Neurol 65, 1091-1095 (2008).
6. Montine, T. J. et al. National Institute on Aging-Alzheimer's Association guidelines for the neuropathologic assessment of Alzheimer's disease: a practical approach. Acta Neuropathol 123, 1-11 (2012).
7. Mirra, S. S. et al. The Consortium to Establish a Registry for Alzheimer's Disease (CERAD): Part II. Standardization of the neuropathologic assessment of Alzheimer's disease. Neurology 41, 479-479 (1991).
8. Mizutani, T. & Kasahara, M. Hippocampal atrophy secondary to entorhinal cortical degeneration in Alzheimer-type dementia. Neurosci Lett 222, 119-122 (1997).
9. Wilhelmsson, U. et al. Redefining the concept of reactive astrocytes as cells that remain within their unique domains upon reaction to injury. Proc Natl Acad Sci U S A 103, 17513-17518 (2006).
